# New Appliances and Things Medical

**Published:** 1903-06-20

**Authors:** 


### NEW APPLIANCES AND THINGS MEDICAL.

[We shall be glad to receive, at our Office, 28 & 29 Southampton Street, Strand, London, W.C., from the manufacturers, specimens of all new preparations and appliances which may be brought out from time to time.]

### PANOPEPTON.

(FAIRCHILD BROTHERS AND FOSTER. LONDON AGENTS: BURROUGHS, WELLCOME AND CO., SNOW HILL, LONDON, E.C.)

WE have on more than one occasion called attention to this excellent predigested food. It consists of the nutritive principles of lean beef and wheat flour, thoroughly cooked, digested, sterilized, and concentrated in vacuo. Containing as it does peptones and carbohydrates which can be absorbed from the digestive tract without further aid from the natural digestive functions of the alimentary canal, its use is clearly indicated when these natural functions are in abeyance. In chronic dyspeptic cases, in cases of gastric ulcer, and high fever, panopeptone will be found to give eminently satisfactory results.

